# Intravital Microscopy of the Mouse Brain Microcirculation using a Closed Cranial Window

**DOI:** 10.3791/2184

**Published:** 2010-11-18

**Authors:** Pedro Cabrales, Leonardo J. M. Carvalho

**Affiliations:** Bioengineering, University of California, San Diego; La Jolla Bioengineering Institute

## Abstract

This experimental model was designed to assess the mouse pial microcirculation during acute and chronic, physiological and pathophysiological hemodynamic, inflammatory and metabolic conditions, using *in vivo *fluorescence microscopy. A closed cranial window is placed over the left parieto-occipital cortex of the mice. Local microcirculation is recorded in real time through the window using epi and fluorescence illumination, and measurements of vessels diameters and red blood cell (RBC) velocities are performed. RBC velocity is measured using real-time cross-correlation and/or fluorescent-labeled erythrocytes. Leukocyte and platelet adherence to pial vessels and assessment of perfusion and vascular leakage are made with the help of fluorescence-labeled markers such as Albumin-FITC and anti-CD45-TxR antibodies. Microcirculation can be repeatedly video-recorded over several days. We used for the first time the close window brain intravital microscopy to study the pial microcirculation to follow dynamic changes during the course of *Plasmodium berghei* ANKA infection in mice and show that expression of CM is associated with microcirculatory dysfunctions characterized by vasoconstriction, profound decrease in blood flow and eventually vascular collapse.

**Figure:**
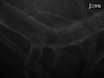


## Protocol

### 1. Craniotomy

A craniotomy in 8-to-10-week old mice needs to be performed in advance as previously described^1^, except that a titanium bar is not placed in the head of the animal. The chronic cranial window is a stable preparation allowing examination of the pial microcirculation even months after being implanted. Usually, we perform our studies 2-3 weeks after the cranial window implantation.

### 2. Intravital microscopy

1. Two-three weeks after craniotomy, body temperature is checked and then the mouse is lightly anesthetized with isoflurane (4% for induction, 1-2% for maintenance). Light anesthesia is used to prevent animal stress and discomfort during the experimental procedure.
2. Animals are then placed in prone position on a stereotaxic frame and the head carefully secured using ear bars. The level is adjusted using the right and left levers. Core body temperature is maintained using a heating pad. Because mice with cerebral malaria develop hypothermia, the temperature settings need to be adjusted for each animal.
3. The cover slip on the cranial window is gently cleaned with a cotton swab moistened with mineral oil and a panoramic picture of the vessels under the window is taken with the help of a stereomicroscope and a digital camera (Nikon Coolpix 995, Japan). The picture is printed, identified and dated and will be used as a map for the measurements of vessel diameter and red blood cell (RBC) velocities.
4. The mouse is then transferred to an intravital microscope stage (customized Leica-McBain, San Diego, CA). A drop of water is placed on the cranial window taking advantage of the well formed by the dental acrylic, focus is adjusted. Measurements are carried out using a 20X (LUMPFL-WIR, numerical aperture 0.6, Olympus) water immersion objective. The same vessels are followed so that direct comparisons to their baseline levels could be performed allowing for more robust statistics for small sample populations. Images are captured using a digital low light high speed camera (Hamamatsu C9300-221, Japan), or low light analogue camera (COHU 4815, San Diego, CA) connected to a VCR tape (JVC, Japan), time stamped (MicroImage Video Systems, Boyertown, PA) and a color monitor (PELCO, Clovis, CA). The tape is properly identified before recording starts, and the time stamp allows identification of the events by documenting the timeframe during which such events were recoded.
5. A first check of the vessels is made to evaluate the quality of the preparation and if the blood is flowing in all vessels. Then, selection of the vessels to be measured is made and it should include venules and arterioles of different diameters (in our mouse pial preparations, most vessels range between 20 and 80μm) and cover different locations within the area exposed by the window. Arterioles and venules can be easily differentiated by checking the direction of the blood flow in the ramifications of the vessel (whether it distributes or collects blood). In our studies, measurements are made in 12 vessels. Precise location of each spot to be measured is annotated in the picture of the pial vasculature.
6. For each spot, the vessel diameter is measured using an image shear device (Image Shear, Vista Electronics, San Diego, CA)^2^. Once the spot is selected, the image of the vessel is aligned in vertical position and the image is sheared until the opposed extremes are aligned and the reading is documented. This value can be translated into micrometers by previous calibration for each specific magnification using a Reticle Calibration Stage Micrometer (Edmund Optics Inc., Barrington, NJ).
7. Each spot is recorded during at least 30 seconds for erythrocyte tracking. For tracking, a sample of blood is collected, and erythrocytes are fluorescently labeled with the carbocyanine dye Dil (Molecular Probes, Carlsbad, CA) and infused through the tail vein to obtain an *in vivo* concentration of ~0.4-0.5%^3^. In animals infected with *Plasmodium berghei* ANKA (PbA) expressing the green fluorescent protein (PbA-GFP, a donation from the Malaria Research and Reference Reagent Resource Center   MR4, Manassas, VA; deposited by CJ Janse and AP Waters), no infusion of labeled cell is necessary. The video images are recorded at 150 frames per second. This rate is set to obtain one to six images of a cell on one video frame for determination of velocities up to 8 mm/s. The video images are digitalized with a personal computer using Adobe Premier 4.0 software, and x-y coordinate data for each cell image are obtained using SigmaScan Pro 4.0 software (SPSS Chicago, IL)^4^. Cell positions are determined manually rather than by image analysis, given that the eye of a trained observer was shown to give a good estimation of the location of the center of a cell, which in general corresponds to the location of maximal fluorescence observed for most cell orientations. Multiple determinations of position and velocity are made for each cell and averaged to obtain mean RBC velocity.
8. When RBC velocity measurements are performed offline, the total observation time for each mouse does not exceed 10-15 minutes minimizing the cardiodepressive effects of anesthesia.
9. Once vessel diameter and RBC velocity measurements are available, calculation of the blood flow in each vessel can be made by using the formula: Q = V x π(D/2)^2^, where Q = blood flow, V = RBC velocity and D = vessel diameter.
10. These procedures can be repeated over time. For instance, in the mouse model of cerebral malaria, we perform daily measurements starting on day 4 of infection. The values obtained every day for each mouse are normalized in relation to day 0 (pre-infection), which is considered to be 100%.
11. In addition to vessel diameter and blood flow measurements, additional evaluations can be performed, for instance improved assessment of perfusion and the analysis of leukocyte and/or platelet rolling and adherence in pial vessels. These measurements are performed with the help of fluorescent-labeled markers. In the case of malaria, we can also detect circulating or adherent parasites by means of using the PbA strain that expresses GFP.
12. For perfusion assessment we use a solution of FITC-labeled albumin (Sigma, St Louis, MO   50μg/mouse), and to quantify leukocyte adherence to pial vessels we use antibodies against the pan-leukocyte marker CD45 labeled with Texas Red (TxR) (Invitrogen, Carlsbad, CA   4μg/mouse). For this, 25μL of the Albumin-FITC (2mg/mL) and 20μL of the anti-CD45-PE antibodies (200μg/mL) are mixed and injected in the pre-warmed tail vein. The mouse can then be imaged immediately, however if leakage measurements will take albumin-FITC is allowed to circulate for 15 minutes. Green fluorescence (518nm) emitted by albumin-FITC and GFP (PbA-GFP pRBC) is captured using and ALPHA Vivid: XF100-2 (Omega Optical, Brattleboro, VT), and anti-CD45-TxR fluorescence (615nm) is exited and captured with a Vivid Standard: XF42.
13. The fluorescent-labeled albumin allows improved visualization of the vascular network, including penetrating vessels, and it is particularly useful in disease states such as cerebral malaria to check for non-perfused or under-perfused vessels. It can also be used to measure vascular leakage^5^.
14. The fluorescent-labeled anti-CD45 antibodies make it easy to identify and quantify leukocyte rolling and adhesion to pial vessels. To avoid bias in quantification, we quantify rolling and adhesion in the same spots pre-defined to measure blood flow. Quantification of leukocyte adhesion is made by counting the number of leukocytes in a 100μm-vessel length. Rolling is quantified by counting the number of leukocytes travelling at a velocity significantly slower than blood velocity in the same 100μm length, during 30 seconds.

### 3. Representative results


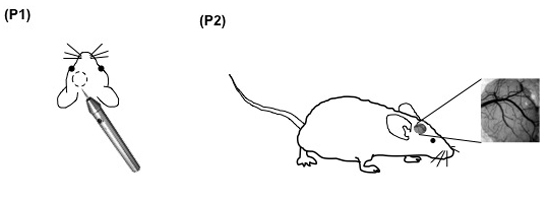
 **Figure 1. (Step 2.6)** Pictures of the mouse pial vascular network accessible through the cranial window.


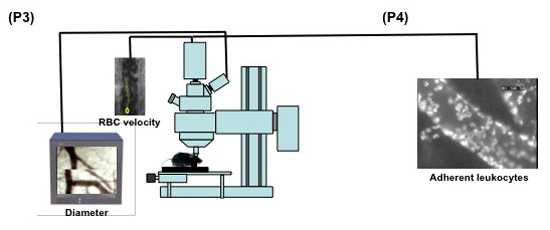
 **Figure 2. (Steps 3.1-3.5)** Schematic display of the set up for intravital microscopy of the mouse pial microcirculation. 1: intravital microscope; 2: 20X water immersion objective; 3: light source; 4a: digital low-light high-speed camera; 4b: analog camera; 5: mouse in the stereotaxic frame; 6: computer monitor; 7: analog shearer monitor showing how the image shearing (arrow) is done to measure vessel diameter.


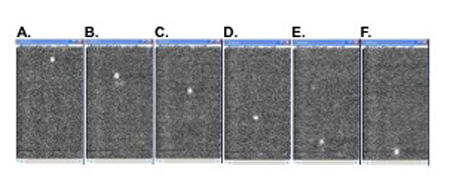
 **Figure 3. (Step 3.6-3.8)** Microvascular red blood cell velocity measurements by cell tracking from high speed fluorescence video recordings. Pictures A to F are sequence images of one pial vessel, showing a single moving RBC crossing the microscopic field delimited by the camera. Manual determination of frame by frame positions of 15 or more cells crossing the field, with its pre-calibrated distance, allows calculation of mean RBC velocity in each vessel with the help of an Excel spreadsheet.


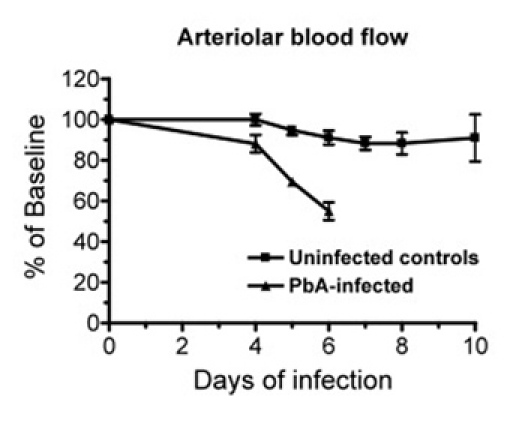
 **Figure 4. (Step 3.9)** Data of a representative experiment showing the changes in pial blood flow over time in *Plasmodium berghei* ANKA (PbA) infected mice (n = 5) and in uninfected control mice (n = 5). Whereas in control mice the pial blood flow is relatively stable over time, PbA-infected mice show a marked decrease in blood flow at the time of cerebral malaria development (day 6). Data are the mean ± SEM.


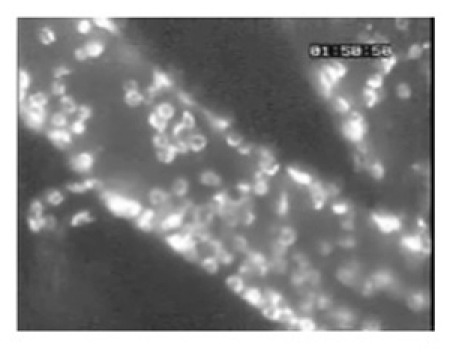
 **Figure 5. (Step 4.3)** A large number of leukocytes adherent to pial vessels of a mouse infected with *Plasmodium berghei* ANKA, as revealed by staining with anti-CD45-Texas Red fluorescent antibodies.

## Disclosures

No conflicts of interest declared.

## Discussion

The intravital microscopy method described here provides a unique and powerful tool for detailed observation of the pial microcirculation in the mouse. It allows singling out individual arterioles and venules and measuring changes of a number of parameters such as vessel diameters, RBC velocities, blood flow, adherence and rolling of leukocytes, platelets and other blood elements, vascular leakage, tissue pH and pO2 and potentially many other applications. The *in vivo* vascular response can be promptly evaluated upon interventions such as drug administration, or during pathological processes. Moreover, the microcirculatory behavior can be dynamically followed up over time. We have used this technology to study the pial microcirculatory changes during cerebral malaria caused by *P. berghei* ANKA in the mouse, and have shown that the neurological syndrome in this model is associated with a microcirculatory dysfunction characterized by reduced cerebral blood flow, vasoconstriction, impaired perfusion and eventually vascular collapse^6^.
